# Potent Anti-Inflammatory Activity of Carbohydrate Polymer with Oxide of Zinc

**DOI:** 10.1155/2014/712312

**Published:** 2014-03-12

**Authors:** Mario Adan Moreno-Eutimio, Nayeli Goreti Nieto-Velázquez, Lorena Espinosa-Monroy, Yessica Torres-Ramos, Araceli Montoya-Estrada, Jorge Cueto, Juan Jose Hicks, Gustavo Acosta-Altamirano

**Affiliations:** ^1^Immunobiology Laboratory, Mexico's Juarez Hospital, Ministry of Health, 07760 Mexico City, Mexico; ^2^Biochemistry Department, National Institute of Perinatology, Ministry of Health, 11000 Mexico City, Mexico; ^3^Health Sciences Faculty, Anahuac University, 52786 State of Mexico, Mexico; ^4^Health Research Policies, Coordinating Commission of National Health Institutes and Highly Specialized Hospitals, 01900 Mexico City, Mexico; ^5^Mexico's Juarez Hospital, Ministry of Health, 07760 Mexico City, Mexico

## Abstract

Pebisut is a biological adhesive composed of naturally occurring carbohydrates combined with zinc oxide (ZnO) initially used as a coadjutant for healing of anastomoses. Likewise some works demonstrated that carbohydrate complexes exerts anti-inflammatory activity and it is widely known that ZnO modulate inflammation. However, the direct effects of Pebisut on isolated cells and acute inflammatory responses remained to be investigated. The present study evaluated anti-inflammatory effect of Pebisut using lipopolysaccharide (LPS) stimulated human mononuclear cells, chemotaxis, and cell infiltration *in vivo* in a murine model of peritonitis. Our data show that human cells treated with different dilutions of Pebisut release less IL-6, IL-1**β**, and IL-8 after LPS stimuli compared with the control treated cells. In addition, Pebisut lacked chemotactic activity in human mononuclear cells but was able to reduce chemotaxis towards CCL2, CCL5, and CXCL12 that are representative mononuclear cells chemoattractants. Finally, in a murine model of peritonitis, we found less number of macrophages (F4/80^+^) and T lymphocytes (CD3^+^) in peritoneal lavages from animals treated with Pebisut. Our results suggest that Pebisut has anti-inflammatory activity, which might have a beneficial effect during anastomoses healing or wounds associated with excessive inflammation.

## 1. Introduction

Anastomotic complications are associated with increased morbidity and mortality after surgical procedures. Pebisut is a biodegradable adhesive mainly composed of vegetal occurring complex polymers combined with ZnO that provides protection during the critical days of wound healing preventing anastomotic leaks [1].

Traditionally biological adhesives have been used to prevent anastomoses leaks and improve the wound healing; the most used adhesives are composed of fibrin based products or collagen patches that have several disadvantages such as infection predisposition and high cost [2]. Despite the importance of protecting healing of the anastomosis surgeons currently do not have effective, safe biological glues that would also reduce the inflammatory process in order to prevent this very serious complication.

Wound healing is a highly regulated process, which involves interactions of extracellular matrix molecules, cytokines, resident cells, and infiltrating cells subtypes. The first goal in the healing process is to achieve tissue integrity and homeostasis [3]. Although inflammation recruits cells for remodeling the damaged, persistent inflammatory stimuli lead to insufficient healing of local tissue [4].

Vegetal polymers of dextrin derivate produced by the hydrolysis of starch obtained from natural products have been described to have anti-inflammatory properties as well as polymers from marine invertebrates composed by fucose, galactose, xylose, and mannose [5–8].

Pebisut is a biodegradable and not toxic natural glue that may be exert anti-inflammatory activity similar to other compounds of natural polymers [7–9]. Besides, Zn component in Pebisut formulation is an essential trace element that plays a crucial role as a cofactor in a number of enzymes and different cellular activities. ZnO is a pharmaceutically acceptable additive that is widely used in dermatology as a wound healing enhancer, because it induces reepithelization, and as a plus exerts anti-inflammatory and antimicrobial activity [10].

Therefore, the present study was designed to test anti-inflammatory effect of Pebisut. Using LPS-stimulated human mononuclear cells (PBMCs) we found that PBMCs treated with the biological adhesive at different dilutions produced less amounts of inflammatory cytokines such as IL-6, IL-1*β*, and IL-8 after LPS stimuli compared with the control treated cells. Additionally, Pebisut lacks chemotactic activity over PBMCs but is able to reduce chemotaxis towards CCL2, CCL5, and CXCL12. Finally, in a murine model of thioglycollate-induced peritonitis, we found reduced numbers of macrophages (F4/80^+^) and T lymphocytes (CD3^+^) in peritoneal lavages from animals treated with Pebisut.

Although the direct effects of Pebisut over inflammatory process* in vivo* remained to be clarified our data showed the direct anti-inflammatory effect of this biological adhesive that may be an advantage compared with the classical fibrin based products used during anastomoses care.

## 2. Materials and Methods

### 2.1. Ethics Statement

The project was approved by the Mexico's Juarez Hospital Scientific Research Committee (composed by Scientific, Ethics, and Bio-security Committees, Project no. HJM 2210/12A). The protocol complied with the Regulations of the General Health Law on Animal Research in Mexico (NOM062-ZOO-1999).

### 2.2. Materials

*Reagents.* Pebisut with patents granted by the United States Patent US 8,252,333, 2012, in the European Union 2,062,602, 2010, in Mexico P.C.T./MX/a/2009/001737, and in Canada number 2661686, 2012, is based in a naturally occurring complex carbohydrate polymer with a metal oxide that includes a zinc ion, is biodegradable, nontoxic, environmental-friendly, and water-soluble, does not require special storage conditions, does not allow microbial colonization, and is produced locally following Good Manufacture Practices.

The antibodies PeCy5-F4/80, Pe-CD3, Pe-CD19, and PerCP-Ly-6G/Ly-6C-GR1 were purchased from BD (Becton Dickinson, San Jose, CA, USA) and human recombinant chemokines from PeproTech (Rocky Hill, NJ, USA). Hank's balanced salt solution was purchased from Invitrogen (Piscataway, NJ, USA) and supplemented with 0.05% endotoxin-free bovine serum albumin from Sigma Chemical (St. Louis, MO, USA). Calcein AM was purchased from Invitrogen. For the chemotaxis assays used chambers from Neuro Probe, Inc. (Gaithersburg, MD, USA), as well as polycarbonate membranes of 5 *μ*m, 25 × 800 mm, with PVP surface treatment.

*Mice.* Six-week-old male C57BL/6 mice were purchased from Harlan-Mexico (Mexico City) and kept at the animal facilities of the Experimental Surgery Unit of Mexico's Juarez Hospital. The animals were cared for in accordance with laboratory practice guidelines recommended by institutional and national regulations.

### 2.3. PBMCs Stimulation

Cells were isolated from blood of healthy human donors by centrifugation through a Histopaque 1077 (Sigma-Aldrich, Poole, UK) density gradient. Then 1 × 10^6^ cells/mL per well in 24 well plates were resuspended in RPMI-1640 (Gibco by Life Technologies, NY, USA) and were stimulated with a mix of 1 : 100 or 1 : 500 of Pebisut alone or with 100 ng/mL of* E. coli* LPS (Sigma-Aldrich, Poole, UK) during 18 hr at 37°C in a 5% CO_2_ atmosphere; after incubation time the supernatants were stored at −70°C.

### 2.4. Nitric Oxide (NO) Measurement

Nitrite was measured by addition of 100 *μ*L of Griess reagent (1% sulphanilamide and 0.1% naphthylenediamine in 5% phosphoric acid; Sigma) to 100 *μ*L of the supernatants. Optical density was read at 540 nm and NO concentration was determined by using the standard concentrations of sodium nitrite (0–100 *μ*M) [11].

### 2.5. Chemotaxis Assay

Chemotaxis assay was carried out using modified Boyden's chambers. Briefly, in experiments using Pebisut, cells were preincubated with different dilutions of the product (1 : 100 and 1 : 500) for 15 min at 37°C prior to the assay. Chemokines (100 ng/mL) were loaded into the lower chamber, which was separated from the cells (10^6^/mL) in the upper chamber by a 5 *μ*m polycarbonate membrane. After 90 minutes of incubation at 37°C, the membrane was removed. The nonmigrating cells were washed away, and membranes were air-dried; the migrating cells were quantified by detecting fluorescence in a Typhoon scanner apparatus (Bio-Rad). The fluorescence detected was used to calculate migration index as the x-fold increase in migration was observed over the control: migration index = fluorescence of migrated cells divided by the basal migration fluorescence (without chemokine).

### 2.6. Thioglycollate-Induced Peritonitis

Mice were injected i.p. with 1 mL of sterile 3% Brewer's thioglycollate broth (TG) (Sigma-Aldrich, Poole, UK) and sterile saline solution was used as a vehicle in control mice. Some animals were treated with 0.2 mL of 1 : 100 dilution of Pebisut in sterile saline solution via i.p. injection 24 hr after TG administration. Mice were ethically sacrificed by CO_2_ inhalation 72 hr after TG injection. Finally the peritoneal cavity was flushed with 5 mL of cold PBS. The collected peritoneal cells were counted and prepared for phenotypic analysis by flow cytometry of macrophages (F4/80^+^), T lymphocytes (CD3^+^), B lymphocytes (CD19^+^), and granulocytes (GR1^+^). The peritoneal fluids were separated from cell pellet and were stored at −70°C for cytokine measurements.

### 2.7. Cytokines Determination

A set of inflammatory human cytokines, TNF-*α*, IL-8, IL-6, IL-12, IL-10, and IL-1*β*, were quantified from the supernatants using bead-based assays (BD Cytometric Bead Array inflammatory; BD Biosciences, San Jose, CA, USA) in accordance with the manufacturer's instructions. Fluorescence from the beads was detected using a BD Accury C6 flow cytometer system (Becton Dickinson, San Jose, CA, USA) and analyzed with FCAP Array Software.

### 2.8. Statistical Analysis

Statistical significance between specific groups was determined using one-way ANOVA with the Bonferroni's post hoc test and statistically significant differences are indicated by asterisks as follows: *P < 0.05, **P < 0.01, and ***P < 0.001. Statistical analyses were performed using GraphPad Prism version 5.0 (GraphPad Software, La Jolla, CA, USA).

## 3. Results

### 3.1. Pebisut Reduces Cytokines Production Induced by LPS Stimuli in Human PBMCs

**Figure 1:**
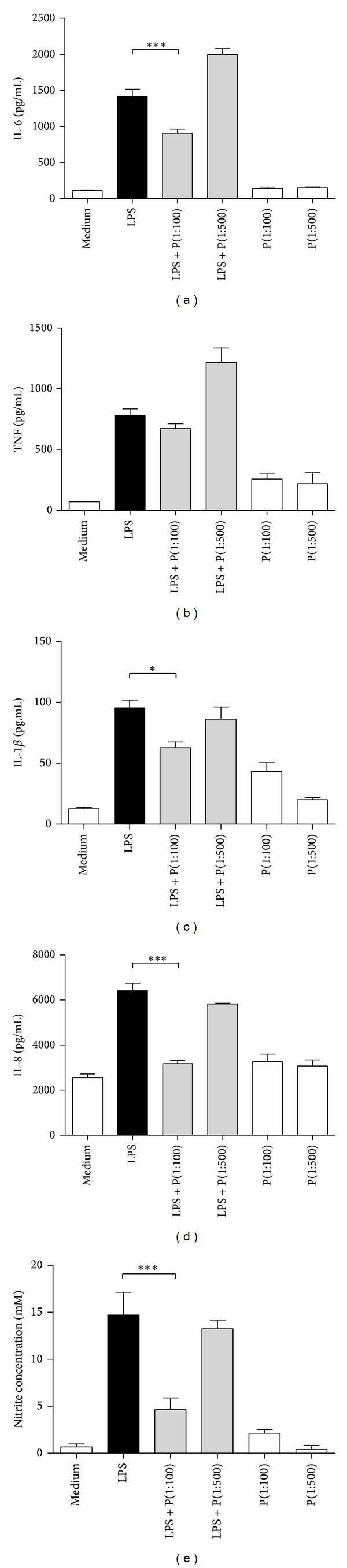
Effects of Pebisut on inflammatory cytokine production induced by LPS. Peripheral blood mononuclear cells were purified and incubated in the presence of Pebisut (1 : 100 or 1 : 500) with or without LPS (100 ng/mL) stimulation during 18 hrs. After incubation, supernatants were collected to determinate cytokine concentration by CBA. (a) IL-6, (b) TNF, (c) IL-1*β*, (d) IL-8, and (e) nitrite. The data are expressed as the mean ± SEM and were analyzed with one-way ANOVA followed by Bonferroni's comparison test (***P < 0.001, **P < 0.01, and *P < 0.05). Data are representative of three independent experiments.

Our data show that Pebisut at a 1 : 100 dilution reduced IL-6 concentration in the supernatant comparing with LPS stimulated cells (Figure 1(a)); the same results were found with IL-1*β* (Figure 1(c)) and IL-8 (Figure 1(d)); no effect was observed on the levels of TNF (Figure 1(b)) and Pebisut at a 1 : 500 dilution did not reduce the levels of IL-1*β*, IL-8 and nitrate release. A slight increase in levels of IL-6 and TNF was observed when stimulated with LPS and Pebisut at a 1 : 500 dilution; this effect was probably due to some component of this dilution that promotes LPS signaling; more research is needed to better understand. Interestingly we observed that Pebisut treatment without LPS stimulation did not have effect in the cytokine production comparing with not stimulated cells (Figure 1). Thus these data show as first time the anti-inflammatory effect of Pebisut* in vitro* in this case using LPS as a stimulus that is a model closely related to some of the innate responses related to inflammation [12].

### 3.2. Inhibition of NO Production Induced by LPS

As shown in Figure 1(e), 100 ng/mL of LPS induces 14.7 ± 4.1 mM nitrate release. The mixture of LPS and Pebisut at a 1 : 100 dilution reduced nitrate production significantly.

### 3.3. Pebisut Reduces Cell Migration under a Chemotactic Stimulus *In Vitro*

We determine PBMCs attraction towards CCL2, CCL5, and CXCL12, which are some of the most important CCR1, CCR5, and CXCR4 ligands. In these assays, we found reduced chemotaxis towards tested chemokines (100 ng/mL) when cells were in presence of Pebisut at a 1 : 100 and 1 : 500 dilution.

**Figure 2:**
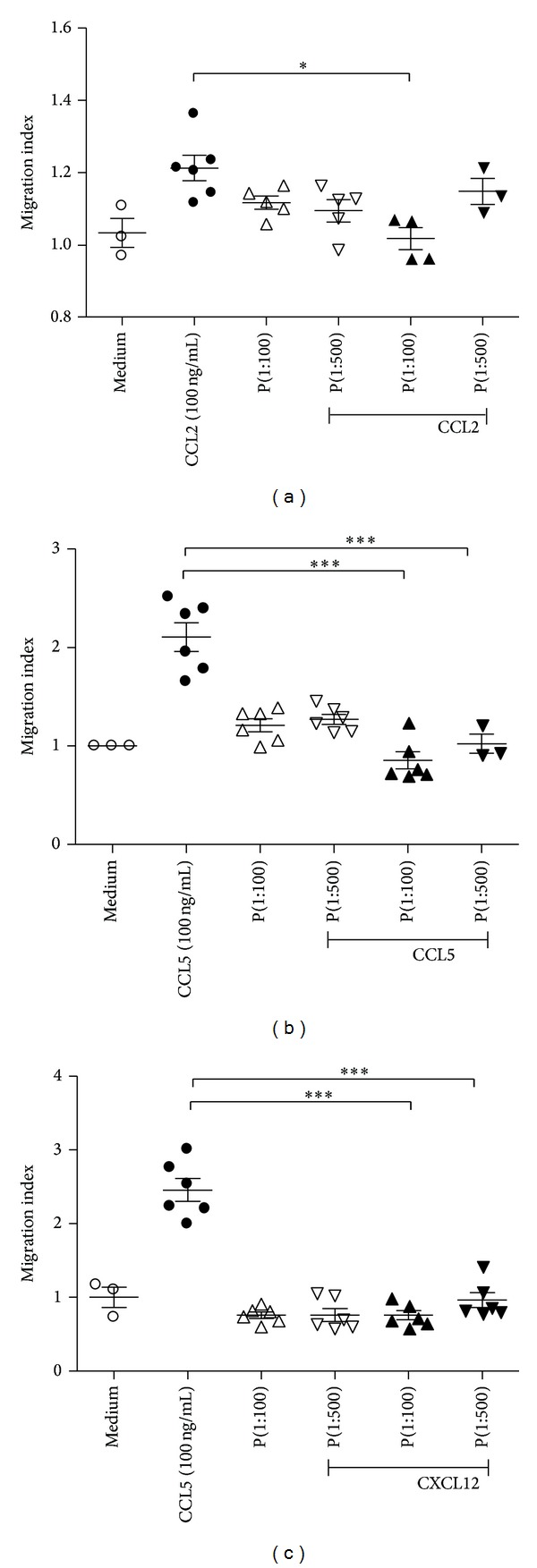
Chemotaxis induced by CCL2, CCL5, and CXCL12 is reduced by Pebisut in PBMCs. Chemotaxis assays with PBMCs were performed using membranes of 5 *μ*m pore, 2 × 10^5^ cells per well were preincubated with Pebisut (1 : 100 or 1 : 500), and 100 ng/mL chemokines were added. (a) CCL2, (b) CCL5, and (c) CXCL12. The data are expressed as the mean ± SEM and were analyzed with one-way ANOVA followed by Bonferroni's comparison test (***P < 0.001, **P < 0.01, and *P < 0.05). Data are representative of three independent experiments.

Pebisut at a 1 : 100 dilution reduced chemotaxis towards stimulus of 100 ng/mL of CCL2 showing significant differences (*P* < 0.05) comparing with the pick of CCL2 migration (Figure 2(a)). Pebisut at a 1 : 100 and 1 : 500 dilution reduced chemotaxis towards stimulus of 100 ng/mL of CCL5 (*P* < 0.001) and CXCL2 (*P* < 0.001) where Pebisut treated cells showed reduced chemotaxis towards mentioned chemokines (Figures 2(b) and 2(c)). Additionally, it is important to mention that Pebisut lacks chemotactic properties over tested cells (Figure 2). Our data suggest that Pebisut reduces the chemotaxis of mononuclear cells induced by tested chemokines that represent the major chemotactic molecules which attract cells at the inflammatory foci, in particular, monocyte/macrophage and lymphocytes chemoattractants such as CCL2, CCL5, and CXCL12.

### 3.4. Pebisut Reduces Recruitment of Macrophages and T Lymphocytes in an Experimental Model of Peritonitis in Mice

**Figure 3:**
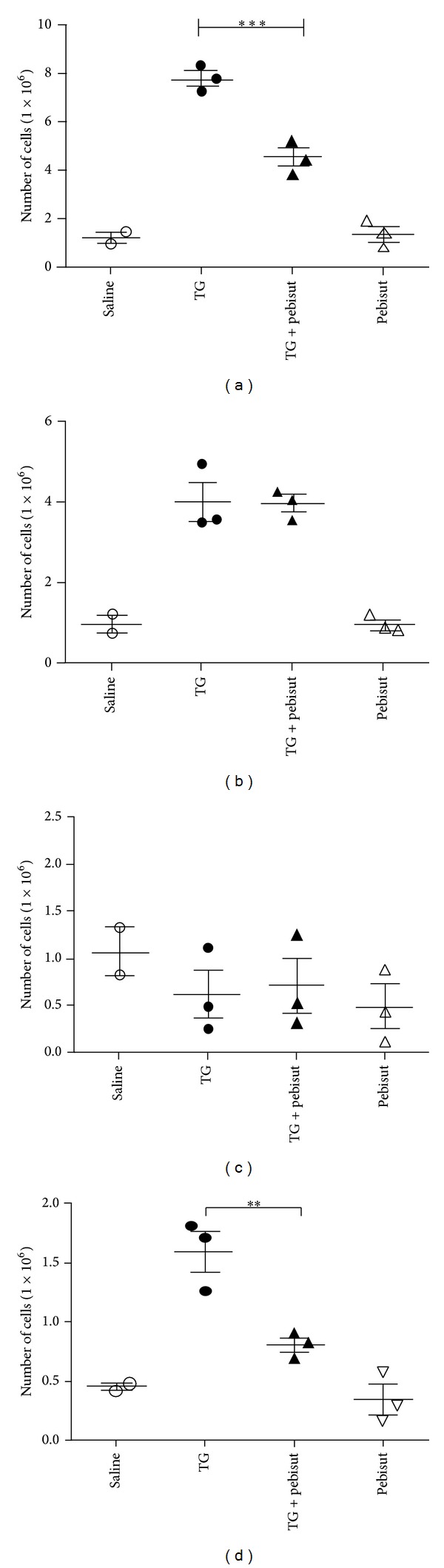
Pebisut reduces the number of macrophages and T lymphocytes in peritoneal lavages. Cells were obtained from peritoneal cavity and analyzed by flow cytometry as previously described. (a) macrophages (F4/80^+^), (b) granulocytes (GR1^+^), (c) B lymphocytes (CD19^+^), and (d) T lymphocytes (CD3^+^). The data are expressed as the mean ± SEM and were analyzed with one-way ANOVA followed by Bonferroni's comparison test (***P < 0.001, **P < 0.01, and *P < 0.05). Data are representative of three independent experiments.

To investigate the hypothesis that Pebisut reduces inflammation by modifying cell function, we used the TG-induced peritonitis model for inflammation. Therefore, we investigated the effect of Pebisut on cell recruitment* in vivo*. In these experiments, we observed that Pebisut injected i.p. 24 hr after injection of TG at a 1 : 100 dilution reduced total number of cells in the peritoneal lavages. Additionally analysis by flow cytometry showed less T lymphocytes (CD3^+^) and macrophages (F4/80^+^) in Pebisut treated group. No significant differences were found in the number of granulocytes and B lymphocytes in either group (Figure 3).

Furthermore, data obtained* in vivo* showed that Pebisut administration after TG injection reduced the number of infiltrating macrophages 72 hours after the irritant injection as well as T lymphocyte in the peritoneal cavity; these data also correlated with our findings* in vitro* where chemotaxis was reduced in presence of Pebisut in human PBMCs towards CCL2 that is a monocyte/macrophage chemoattractant similar to CCL5 for lymphocytes.

## 4. Discussion

We previously describe Pebisut as a biological adhesive which prevents anastomoses leaks; it is a FDA approved additive used in dermatology and several pharmaceutical products [1]. Our previous data demonstrated that Pebisut increases resistance and protection during critical days of the anastomoses wound healing and now is in Phase III of clinical trials. Additionally, Pebisut has been used to treated chronic venous leg ulceration (data not published) that are typical lesions with impaired healing because of a persistent exacerbated chronic inflammatory process.

Pebisut, a biodegradable and nontoxic glue which does not require special storage conditions and does not allow microbial colonization, represents a new generation of biological adhesives with several advantages comparing with the classic fibrin based products used as an adjuvant to prevent anastomoses leaks [1].

Complications of anastomosis following colorectal surgery are a worldwide problem ranged from 4 to 26% producing high mortality and morbidity rates; in addition leaks represent high cost if a second surgery is required [2]. Different procedures have been used to prevent leakages, mainly fibrin derivatives, reinforcement of the staples lines, omentoplasty, and so forth [1, 13].

Besides, exacerbated inflammation linked with abnormal healing leads to infection, fibrosis and cancer [4]. Inflammation is a complex process regulated by several cells and soluble factors that is recognized as a key step to repair tissue injuries [4]. During wound healing acute inflammation is necessary to repair the damage; this inflammation is characterized by spatial and temporal waves of specific subsets of cells and soluble factors [4]. Furthermore, disregulated inflammation is a hallmark of noncorrectly healing wound. Therefore, the present work demonstrated the anti-inflammatory activity of Pebisut using acute inflammation models.

To mimic acute inflammatory states on tissues we use LPS that is a recognized model to activate innate inflammatory responses via TLR4. LPS is a derived gram-negative component of bacterial wall that is a potent activator of innate immune responses [12]. LPS binds to surface TLR4 molecules, triggering the secretion of various inflammatory factors, which contribute to the pathophysiology of septic shock and other immune disease.

Our data using PBMCs demonstrated that Pebisut diluted 1 : 100 reduced IL-1*β* secretion induced by LPS stimuli (Figure 1). In this regard IL-1*β* is an inflammatory cytokine which plays a key role in inflammation and wound healing; several works have demonstrated that blocking IL-1*β* receptor might help to control incision pain after surgery and suggest that topical administration of IL-1*β* may be useful for enhance healing of the wound because it stimulates fibroblast proliferation and the synthesis of fibronectin, collagen, metalloproteases (MMPs), and tissue inhibitors of metalloproteases [4, 14]. IL-1*β* also promotes the production of profibrotic cytokines, including TGF-*β* and IL-6. Our data with IL-1*β*, suggest that this cytokine acts regulating wound healing but its role may depend on the type of injury studied.

The same results were found with IL-6 (Figure 1(c)); this cytokine has crucial roles in inflammation, particularly at the early phase because it regulated leukocyte recruitment to the inflammatory foci and induced fibrotic changes that improve healing [4, 15]. However, IL-6 overexpression is also related with the pathogenesis of several types of skin diseases, including psoriasis and systemic lupus [15].

Additionally we also found lower levels of IL-8 (Figure 1(d)) in PBMCs treated with Pebisut and stimulated with LPS. IL-8 is a chemokine (CXCL8) that represents a selective and specific group of neutrophil chemoattractants that are crucial during the first wave of responses during inflammation, but during wound healing IL-8 increases reepithelialization by stimulating keratinocyte proliferation and migration but also elevated levels of IL-8 contribute to retarded wound closure [16–18].

Furthermore, Pebisut lacks activity over TNF-*α* secretion after LPS stimuli (Figure 1). TNF-*α* is a potent cytokine exerting critical beneficial activities in immune regulation and host defense, as well as hazardous proinflammatory and cytotoxic function [19, 20]. In this regard activation of TLR4 induces secretion of inflammatory cytokines such as TNF-*α*; these cytokines are released in response to several intracellular pathways that activate gene transcription [12]. For TLR4 it is well known that Myd88 pathway leads to nuclear translocation of NF*κ*B that is the master transcription factor of inflammatory genes [21]. Our data shows that Pebisut has no effect on TNF-*α* secretion but a strong effect on the production of IL-8 and IL-6; some works demonstrated that TNF-*α* secretion different to other mentioned cytokines depends on some specific factors such as IRAK4 that is not required for IL-6 secretion [19]. But further experiments are needed to know the Pebisut effect over specific intracellular pathways related with specific cytokines.

We also observed that Pebisut treatment without LPS stimulation did not have effect with the basal cytokine production comparing with not stimulated cells (Figure 1). This data showed that Pebisut does not produce inflammation but interestingly exerted anti-inflammatory properties related with cytokine secretion after LPS stimuli; however, we have to further investigate the mechanism.

In addition we looked into the effect of Pebisut in the chemotaxis of PBMCs using* in vitro* assay. Our data demonstrated that Pebisut reduced migration towards chemokines such as CCL2 that is a major monocyte/macrophage chemoattractant via CCR2 activation (Figure 2) [22]. We suggest that chemotaxis is reduced in presence of Pebisut as a consequence of mechanical barriers, which impaired chemokine receptor interaction, but we do not discard a different molecular mechanism. The same result was found with CCL5 that is a ligand of CCR5 expressed en T lymphocytes; this chemokine has been recognized as an important mediator during healing [23, 24].

Likewise we tested CXCL12 that is a CXCR4 ligand and similar to CCL tested chemokines we found chemotaxis reduction when cells were preincubated with Pebisut (Figure 2). Although we just tested a few chemoattractants we suggested that chemotaxis impairment in presence of Pebisut is not a selective mechanism and would be more related with mechanical impediment as we explain. Our data also show that Pebisut does not induce chemotaxis in the tested cellular system.

In order to elucidate if Pebisut activities over inflammatory cytokine secretion and chemotaxis observed* in vitro* were also exerted* in vivo* we use a TG peritonitis model that has defined sequential steps starting with the influx of PMN and monocytes (1–4 h) and followed by a macrophages infiltrate (40–72 h) recruitment in the peritoneal cavity. Increased cytokine levels in the peritoneal fluid accompany the inflammation [25].

Using TG-induced peritonitis model we found that Pebisut injected i.p. 24 hours after injection of TG at a 1 : 100 dilution reduced total number of cells in the peritoneal lavages comparing with the control treated mice. Analysis by flow cytometry showed less T lymphocytes (CD3^+^) and macrophages (F4/80^+^) compared with the control group and no significant differences were found in the number of granulocytes and B lymphocytes in either group (Figure 3). 72 hours after TG injection cell infiltration in the peritoneal cavity is mainly composed of macrophages but we do not investigate shorter times in the inflammatory course that should be important to determinate the effect of Pebisut over the neutrophils pick; however, at the analyzed point Pebisut administration does not produce any effect over the number of Gr1^+^ cells obtained from peritoneal lavages but interestingly in our* in vitro* model of LPS stimulation using PBMC we found important reduction in IL-8 release in cells preincubated with Pebisut; in this regard IL-8 is one of the most important neutrophil chemoattractants that in the peritonitis model has been described as very important; in the first wave of infiltrating neutrophils this chemokine is mainly produced by resident macrophages of the peritoneal cavity [25].

Importantly Pebisut lacks chemoattractant properties over analyzed cells when it was injected in the peritoneal cavity of mice, similar as was found in* in vitro* chemotaxis assays where Pebisut did not produce chemotaxis in PBMCs.

However, the mechanism related with anti-inflammatory activity exerted by Pebisut remains to be elucidated; it is well known that some of its components such as ZnO have a widely described activity as reepithelialization agents [10, 26].

Pebisut had important anti-inflammatory activity in acute inflammatory stimuli induced by LPS reducing some of the most important inflammatory cytokines released after TLR4 activation. Besides Pebisut exerts anti-inflammatory activity* in vivo* sterile peritonitis model where we basically observed reduced numbers of macrophages and T lymphocytes in mice treated as well as reduced chemotaxis* in vitro* towards chemokines linked with monocyte/macrophage and lymphocytes migration in human PBMCs. These findings are important, because although initial inflammatory process is essential for the resolution of inflammation and tissue repair that permits normal healing and remodeling. It is also very important to limit and control inflammatory process than significantly interfere with the healing process [4, 27].

Altogether our data demonstrated that Pebisut represents a new generation of biological adhesives with several advantages comparing with the classic fibrin products and is a low cost product with important benefits to prevent anastomosis leaks and improve healing process in impaired wound healing related with excessive inflammation.
